# Development and psychometric testing of an observation-based assessment tool focusing on work-related stressors among health professionals: the STRAIN-External Observation of work Stressors

**DOI:** 10.1186/s12995-020-00275-y

**Published:** 2020-08-26

**Authors:** Karin A. Peter, Esther Stadelmann, Jos M. G. A. Schols, Ruud J. G. Halfens, Sabine Hahn

**Affiliations:** 1 Department of Applied Research & Development in Nursing, Bern University of Applied Sciences, Murtenstrasse 10, 3008 Bern, Switzerland; 2 University Hospital of Zurich, Zurich, Switzerland; 3 Department of Health Services Research, Focusing on Value-based Care and Ageing, CAPHRI - Care and Public Health Research Institute, Maastricht University, Maastricht, The Netherlands; 4 Department of Family Medicine, CAPHRI - Care and Public Health Research Institute, Maastricht University, Maastricht, The Netherlands; 5 Department of Health Services Research, CAPHRI - Care and Public Health Research Institute, Maastricht University, Maastricht, The Netherlands

**Keywords:** Work stressors, Observation, Health professionals, Healthcare sector, Observation-based assessment

## Abstract

**Background:**

Health professionals are especially affected by various stressors in their daily work, such as a high workload, physical and emotional challenges. The aim of this study was to develop and test the validity, reliability and usability of an observation-based instrument designed to assess work stressors in the healthcare sector.

**Methods:**

Using a cross sectional design, 110 health professionals were observed during one entire shift by an external observer. Factor analysis was used to test construct validity, Cronbach’s alpha to test internal consistency and correlations using Kendall’s Tau were computed to test for convergent validity.

**Results:**

For 9 out of 10 tested scales the results showed a one-factor solution for all observation scales (explained variance ranged from 55.5 to 80.2%), satisfactory reliability (Cronbach’s alpha between .67 and .92), sufficient usability and satisfactory convergent validity.

**Conclusions:**

The newly developed STRAIN-EOS, an observation-based assessment tool designed to assess stressors specifically in the healthcare sector, was shown to be potentially useful. However, further refinement and testing is necessary before it can be widely used.

## Abstract (de)

**Hintergrund:**

Gesundheitsfachpersonen sind bei ihrer täglichen Arbeit von diversen Stressoren am Arbeitsplatz betroffen, wie z.B. eine hohe Arbeitslast, physische oder emotionale Anforderungen. Ziel dieser Studie war es, ein beobachtungsbasiertes Instrument zur Erfassung solcher Stressoren im Gesundheitssektor zu entwickeln und auf seine Validität, Reliabilität und Anwendbarkeit zu testen.

**Methode:**

Mittels Querschnittsdesign wurden 110 Gesundheitsfachpersonen während einer gesamten Schicht von einer/einem externen Beobachter (in) begleitet. Die Konstruktvalidität wurde anhand einer Faktoranalyse überprüft sowie Cronbach’s Alpha Werte zur Überprüfung der internen Konsistenz und Kendall’ Tau zur Überprüfung der Konvergenzvalidität berechnet.

**Resultate:**

Für 9 von 10 getesteten beobachtungsbasierten Skalen zeigten die Ergebnisse eine Ein-Faktoren-Lösung (die erklärte Varianz reichte von 55.5% bis 80.2%), zufriedenstellende Reliabilität (Cronbachs Alpha zwischen .67 und .92), Anwendbarkeit und zufriedenstellende Konvergenzvalidität.

**Schlussfolgerungen:**

Das neu entwickelte STRAIN-EOS, ein beobachtungsbasiertes Assessment Tool zur Bewertung von Stressoren speziell im Gesundheitssektor, hat sich als potentiell nützlich erwiesen. Für eine breite Anwendung ist jedoch eine weitere Verfeinerung und Überprüfung des Instruments notwendig.

## Background

Stress at work is becoming increasingly problematic, with one in six European employees reporting chronic stress-related health problems [1]. Health professionals are particularly affected by long working hours and high workloads, shift work with consequences for their work-private life balance, understaffing, emotional demands through confrontation with suffering, death or aggression at work as well as varying physical challenges [2–4]. Stressors at work usually originate in aspects of the design and management of the work, and in the social and organizational contexts. They have a potential for adverse psychological, physical or social outcomes for employees [5]. Besides physical and mental health problems, stress can also negatively influence job satisfaction, intention to leave the job and safety at work [6–9].

Over the last few decades, various approaches and methods have been developed and used to assess stressors at work. Most notable among these are self-report questionnaires and observation- or situation-based measures [10], each of which has its strengths and weaknesses [11]. While self-report questionnaires are most commonly used due to their simple, cost-saving application and the possibility of obtaining large data samples, observation-based instruments seem to provide a more independent view of possible stressors at work [12]. The most common observational instruments focus on job and task analysis (e.g. work function, tasks), company analysis (company stress diagnosis, e.g. CANEVAS,) or are observation-based checklists of work stressors [5, 13].

Previous studies have shown that it is not easy to objectively capture stressors in the workplace. Many aspects of strain are difficult to observe, being internal states (e.g. emotional pressure, inability to cope, perceived lack of support) and mental processes [12, 14]. Also, associations between objectively identified stressors and the health outcomes of employees are usually weaker than employees’ subjectively assessed stressors [5]. In addition, external observers are individuals with their own experiences, perceptions and memories, all of which can contribute to subjective bias [10, 15]. It should also be noted that the convergence between different measures of stressors, for example subjectively and objectively assessed stress indicators, is generally rather low, ranging mostly between 10 and 30% [5, 10]. Objectively assessed stressors should, therefore, not be seen as ‘the true reality’ but rather as a complement to other measures (e.g. subjectively assessed stressors) providing further insights into the current work situation with an ‘objective’ external view [10].

Moreover, external observations can be costly, so that external observers are usually restricted in observation time, which in turn means that only a limited range of working hours/participants can be observed [15]. This can result in the observer compensating for any missing data by using predominant information (e.g. the halo-effect) or common stereotypes related to the observer’s interpretation and level of knowledge [10]. In addition, the presence of external observers may influence the behaviour of the employees, e.g. due to personal characteristics of the observer (personal reactivity) or simply because they are being observed (procedural reactivity) [15, 16].

Observational instruments can also be difficult to apply in the health sector, due to its 24-h operation or unforeseen events. Health sector studies employing observational methods therefore mostly focus on a specific topic (e.g. workload, teamwork, hierarchies) [17–20] using activity and/or work analysis procedures [21, 22], or else apply instruments that have not been developed and tested specifically in the healthcare sector [13]. Observation-based assessment tools that focus on various possible stressors in the healthcare sector are however clearly necessary, and can be used as an alternative or complement to commonly used self-assessment tools.

The aim of this study, therefore, was to develop and test a new observation-based assessment tool that more closely fits working conditions in the healthcare setting and is capable of assessing a number of work-related stressors. The newly developed observation-based tool was then tested for its construct validity, reliability, convergent validity and usability in the healthcare sector.

## Method

### Design

This study is part of the national **STRAIN** project, “work-related **str**ess **a**mong health professionals **in** Switzerland”. The STRAIN project combines data on stress at work from different data sources. First, health professionals’ self-reports regarding work stressors, stress reactions and long-term consequences from more than 160 participating Swiss health organisations using the STRAIN Questionnaire [23, 24] have been collected three times (2017/2018, 2019, 2020). Second, parallel to the health professionals’ self-assessments, relevant key figures on work-related stress are collected in the participating organisations (e.g. absenteeism, turnover-rates). Third, external observers were used to provide an additional perspective on work-related stress. Thus, this study aimed to develop and test an observation-based assessment tool using a cross-sectional study design, the psychometric properties of which were tested in the healthcare setting. The observations were conducted by external observers and the assessment instrument was called **STRAIN-EOS** (**STRAIN** - **E**xternal **O**bservation of Work **S**tressors).

### Development of the STRAIN-EOS assessment tool

**Fig. 1:**
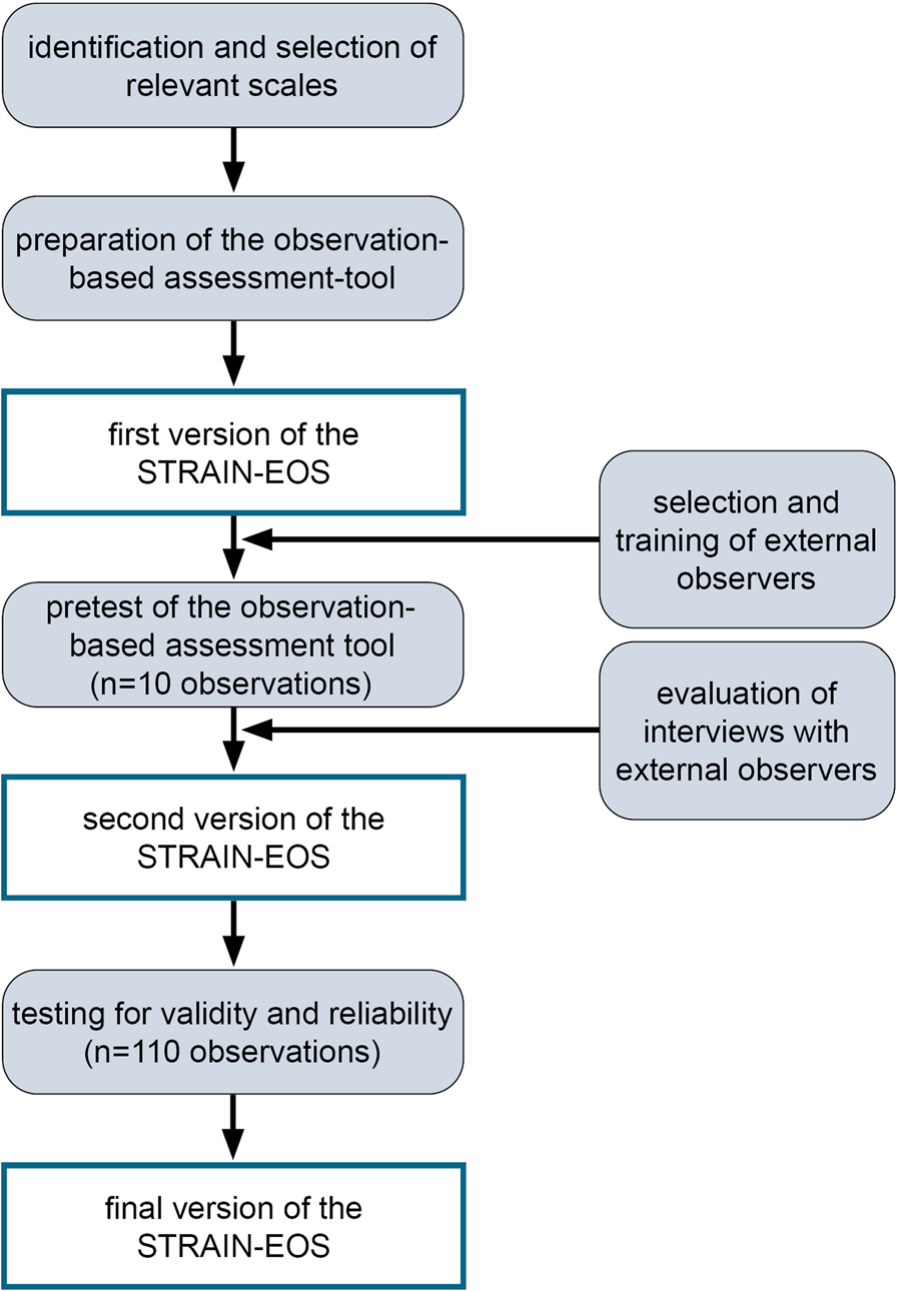
Development steps of the STRAIN-EOS questionnaire

The STRAIN-EOS was developed (see Fig. 1) on the basis of the STRAIN-Questionnaire (employees’ self-reports), using standardised, validated and reliable self-assessment scales which, according to previous studies, are also externally observable [5, 10, 25]. Scales were selected from the German version of the Copenhagen Psychosocial Questionnaire (COPSOQ) [26–29] and the 6th European Working Conditions Survey (EWCS) [30]. According to the COPSOQ, scales were selected based on the thematic fields: a) demands at work, b) work organisation and content, and c) social relation and leadership. The demands at work scales included questions on quantitative, sensorial and physical demands with a response option on a five- or seven-point Likert scale. A high score in these scales indicates a high risk for work-related stress. The scales on work organisation and content are based on questions about possibilities for development, influence at work and degree of freedom at work. Response options are on a five-point Likert scale (always to never). A low score in these scales indicates a high risk for work-related stress. The scales in the social relations and leadership group include questions on predictability, social support, social community, rewards, unfair behaviour and on social relations at work. A low score indicates a high risk for work-related stress. The psychometric properties of these self-report scales had shown good reliability (Cronbach’s alpha > 0.7) and satisfactory construct and criterion validity in previous testing [29, 31].

In a second step, the items of each scale were reformulated so that they could be used as an observational assessment instrument and still be comparable with the underlying self-report scales. Therefore, the same number of items were used as in the self-report scales, and care was taken to ensure that the wording in the STRAIN-EOS items were similar to that used in the self-report (e.g. “Does the observed person have to work very fast?”, for external for observers and “Do you have to work very fast?” for self-reports). Furthermore, a few items for the external observers were added, for example the date of observation, the shift observed, area of work, profession and role of the observed person. One additional question concerning the overall perceived workload in the observed shift was added (according to LEP-AG [32]) to test the reactivity of the observation scales: if an external observer perceived the overall workload as high, this should also result in a higher ranking on observed demand scales. To obtain information about the usability of the STRAIN-EOS, external observers had the opportunity to comment on items (e.g. those which were not observable, difficult to understand). Furthermore, the number of missing values was used as an indication of the STRAIN-EOS’s usability.

In a third step, we pre-tested a first version of the STRAIN-EOS by studying 10 observations in a general hospital setting. The aim was to determine whether all items could be captured by external observers during an entire shift of 9–12 h. Pre-testing indicated that the two scales on ‘rewards’ and ‘unfair behaviour’ were too difficult to assess (not observable in 3 to 9 of the 10 cases). The item ‘the observed person has to do overtime’ (always – never) of the scale on ‘quantitative demands’ was also not observable during one shift. Since all other items were assessable during one shift, these items were excluded from the STRAIN-EOS. Table 1 presents the resulting STRAIN-EOS.

**Table 1:** Content of the STRAIN-EOS (second version)

STRAIN-EOS questionnaire (second version)		items	response option	content
**demographic information**				
general information	in-house developed single items	4	multiple	demographic information on the observed person, date of observation, area of work
**framework conditions at work**				
observed shift	in-house developed single items	3	multiple	hierarchical position, details of the observed shift
**demands at work**				
observed quantitative demands	in-house developed scale according to COPSOQ 2005^a^	6	5-point Likert scale	(1) workload is unevenly distributed; the observed person (2) has to work very fast, (3) does not have time to complete all work tasks, (4) gets behind with his/her work, (5) can take it easy and still do his/her work^e^, (6) does have enough time for his/her work tasks^e^
observed sensorial demands	in-house developed scale according to COPSOQ 2005^a^	5	5-point Likert scale	work of the observed person demands (1) a great deal of concentration, (2) very clear and precise eyesight, (3) controlling his/her movements, (4) constant attention, (5) a high level of precision
observed physical risks	in-house developed scale according to EWCS Q30^c^	4	7-point Likert scale	job of the observed person involves (1) tiring or painful positions, (2) lifting or moving people, (3) carrying or moving heady loads, (4) repetitive arm or hand movements
**work organization and content**				
observed possibilities for development	in-house developed scale according to COPSOQ 2005^a^	7	5-point Likert scale	(1) variety of work, (2) work of the observed person demands a high level of skill or expertise, the observed person (3) has to do the same thing over and over again^e^, (4) work of the observed person requires taking the initiative; the observed person (5) has the possibility to learn new things through his/her work, (6) can use his/her skills or expertise, (7) has the opportunity to develop his/her skills through work
observed influence at work	in-house developed scale according to COPSOQ 2005^a^	10	5-point Likert scale	(1) other people make decisions concerning his/her work^e^, (2) the observed person has a large degree of influence concerning his/her work; observed person has influence on (3) how quickly, (4) when, (5) what (6) how to do his/her work, (7) the amount of work, (8) who to work with, (9) his/her work environment, (10) the quality of his/her work
observed degree of freedom at work	in-house developed scale according to COPSOQ 2005^a^	4	5-point Likert scale	observed person can (1) decide when to take a break, (2) decide when to take his/her holidays, (3) leave work to chat with a colleague, (4) leave work for short private business
**social relations and leadership**				
observed predictability	in-house developed scale according to COPSOQ 2017^b^	2	5-point Likert scale	observed person (1) is well informed in advance, e.g. about important decisions, changes or plans for the future, (2) receives all information needed to do his/her work well
observed social support	in-house developed scale according to COSPOQ 2017^b^	4	5-point Likert scale	observed person (1) gets help and support from colleagues or (2) the immediate superior if needed, (3) colleagues or (4) the immediate superior listen to his/her work-related problems
observed social community	in-house developed scale according to COPSOQ 2005^a^	3	5-point Likert scale	(1) good atmosphere and (2) good cooperation between observed person and colleagues, (3) observed person seems to be part of a community at his/her work
observed social relations	in-house developed scale according to COPSOQ 2005^a^	2	5-point Likert scale	observed person (1) has the possibility to talk to his/her colleagues during work, (2) works isolated from his/her colleagues^e^
**subjective assessment of perceived stress-level during shift**				
observer’s estimated level of work-related stress	in-house developed single items based on Leistungserfassung in der Pflege (LEP®)^d^	1	7-point Likert scale	observer’s estimated level of work-related stress related to his/her observed shift
**questions about non-observable items**				
reasons for non-observable items	in-house developed single items	1	open	documentation of reasons for non-observable items during the observation

^a^Nübling et al. (2005) [29], ^b^Nübling et al. (2017) [28], ^c^Eurofound (2015) [30], ^d^LEP-AG (2017) [32], ^e^reversed scored item

### Calculation of the sample size

A sample size calculation using a Monte Carlo method was performed and Cronbach’s alpha was computed for various sample sizes, using an 8-item scale with a response option on a five-point Likert scale originating from COPSOQ as a proxy. Therefore, datasets on this 8-item scale of varying sizes (10, 50, 100, 150, 200, 300, 500) were generated based on averages and ICCs retrieved from previous results on COSOQ [29]. The accuracy of the Cronbach’s alpha for a given sample size was estimated based on 5′000 such simulated datasets. The analyses indicated that an accuracy of 0.1 points is achieved with approximately 100 observations (95% confidence interval); therefore, a sample size of 100 to 110 participating health professionals was targeted.

### Recruitment of health professionals

Since the participation of organisations as well as individual health professionals was on a voluntary basis, this study is based on a convenience sample using the authors’ professional network. In total, two acute care hospitals, two nursing homes, one home care organization and two psychiatric hospitals, all located in the German-speaking part of Switzerland, declared themselves willing to participate. Health professionals from various disciplines in these organizations (e.g. nurses, medical-technical-therapeutic-professionals, physicians) who had direct patient contact, were working part- or full-time and working on all shifts were invited to participate. They received a written study flyer with detailed information on the study. Health professionals willing to participate in the study had the option to send an e-mail directly to the research team with more information regarding the shift and date for a possible observation.

### Recruitment and training of external observers

Eight external observers with a professional background in the healthcare sector were recruited through advertisement. These external observers were aged between 22 and 40 years, were female, had professional training in nursing, physiotherapy or psychology, and had professional experience ranging from 2 to 19 years. All external observers were given a standardised training session of 10 h. During this training, external observers received information on how to behave during the observations and discussed the comprehensibility and interpretation of each item included in the STRAIN-EOS assessment tool.

### Interobserver reliability

Since it was not possible for more than one external observer to accompany the observed health professional during the shift (requested by participants/organizations / patients), interobserver reliability was tested using 10 video sequences of various health professionals at work in order to represent reality in practice as well as possible. All external observers watched the same video sequences (30–50 min) and assessed the observed stressors separately, using the STRAIN-EOS. The mean intraclass correlation coefficient (ICC, “1 - each target is rated by a different set of k judges, randomly selected from a larger population of judges”, PE Shrout and JL Fleiss [33]) for all items included in an observation scale varied between 0.05 (scale on predictability) and 0.54 (scale on quantitative demands).

### Data collection

Data was collected between December 2017 and May 2018. Participating health professionals were observed by one external observer for an entire shift or working day (9 to 12 h). External observers were permitted to talk to the health professionals they observed, but not to support or help them with their work. The external observers took notes during the observation period and completed the questionnaire (10–15 min) at the end of the shift/working day. Data was collected in acute care hospitals (inpatient medical, surgical and rehabilitation wards, emergency department, physiotherapy, operating room), psychiatric hospitals (general psychiatric wards and forensic), nursing homes and a home care organisation.

### Analyses

Data was analysed using SPSS 25®. For data analysis, all Items were transformed and standardised on a value range from 0 to 100 points (0 being the minimum value, 100 the maximum). The analysis procedure (construction and calculation of scales) was carried out according to the underlying self-report scales [26, 30]. If fewer than half of the questions in a scale had been answered, no average score was calculated [26].

The final STRAIN-EOS is supposed to deliver an average score per scale as well as per thematic field according to the COPSOQ self-report scales [27]. Therefore, an exploratory factor analysis (FA) with orthogonal rotation (varimax) and a listwise deletion of missing cases for each scale and each thematic field (a) demands at work, b) work organisation and content, c) social relations and leadership) was performed to check the one-dimensionality of each scale as well as the possible cross-loading of items (> 0.5). Sampling adequacy for FA was measured by the Kaiser-Meyer-Olkin measure. Second, items were reduced in a stepwise manner using the Cronbach’s alpha value and poor factor loadings in the factor analyses of items as an indicator. We computed the final Cronbach’s alpha values as a test for the reliability of the scales. Third, convergent validity was tested, for which (1) Kendall’s tau-b correlations (most scales were skewed) were computed by combining the additional question about observers’ overall perceived workload in the observed shift compared to their ratings in the STRAIN-EOS, and (2) the mean values of the STRAIN-EOS were graphically compared with the STRAIN questionnaire from the first STRAIN measurement 2017/2018 (8112 self-reports of health professionals [24]).

Finally, the usability of the instrument was tested by descriptively analysing missing values, along with a qualitative content analysis of observers’ comments on questions which were not observable, or which were difficult to understand.

## Results

### Description of the external observation sample

In total, 110 external observations of health professionals were included. External observations were conducted in a psychiatric hospital (*n* = 36), a nursing home (*n* = 30), home care situations (*n* = 24) or an acute care hospital (20). A total of 55% were registered nurses (*n* = 60), 37% nurse’s aides (*n* = 41), 6% medical-technical-therapeutic professionals (*n* = 7) and 2% physicians (n = 2). Observations were conducted during a day shift (63%, *n* = 69), an evening shift (24%, *n* = 26), a night shift (4%, n = 4) or another shift form, e.g. a divided shift (9%, *n* = 11). Most observations were conducted during weekdays (Monday = 20 observations, Tuesday = 20, Wednesday = 20, Thursday = 15, Friday = 18, Saturday = 9, Sunday = 8). Overall, external observations encompass 996 h (including individual break times of usually 36 min for a shift of 9 h), this includes 543 h of observation for registered nurses, 369 h for nurse’s aides, 63 h for medical-technical-therapeutic professionals and 21 h for physicians.

### Construct validity

**Fig. 2:**
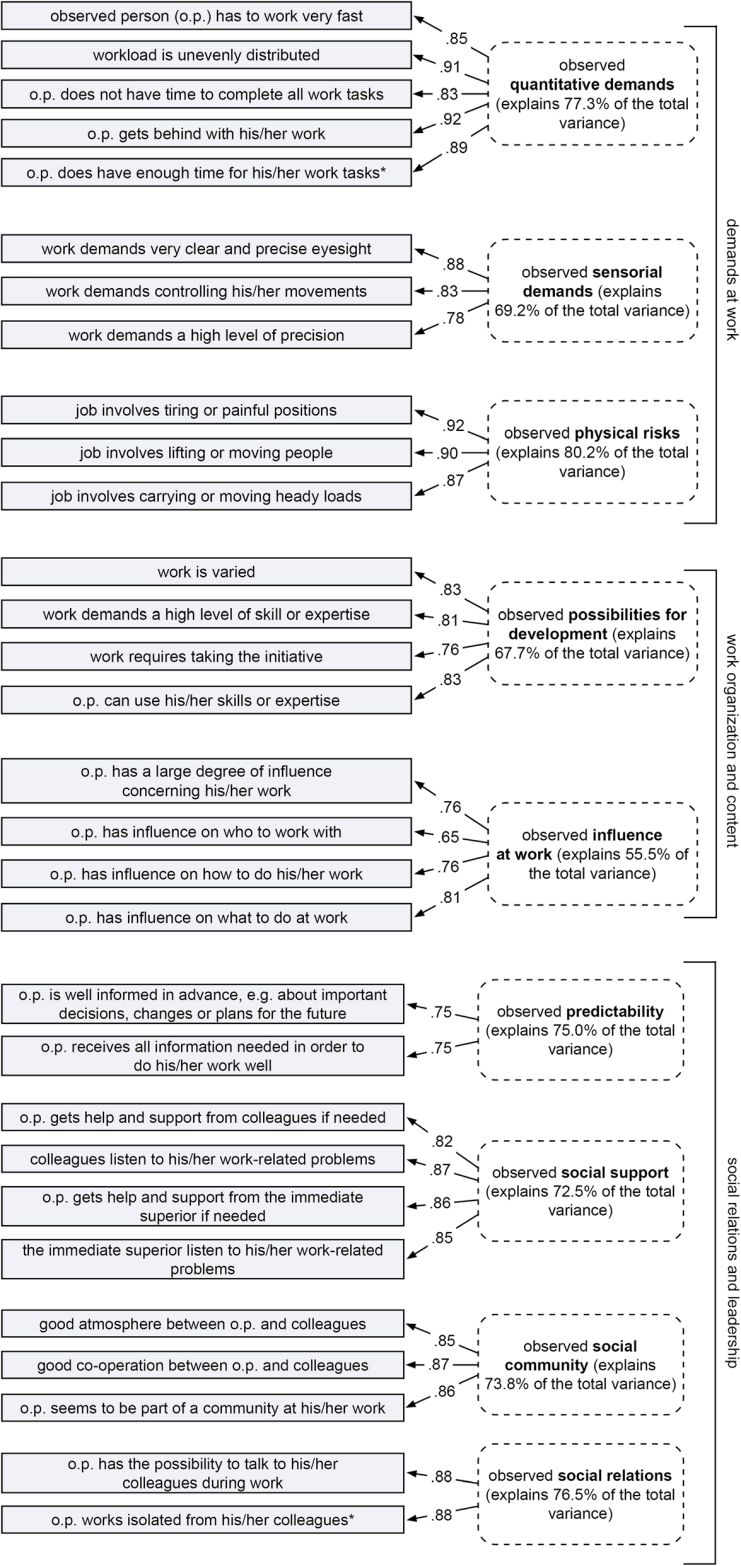
Items, factor loadings and explained variance of the final STRAIN-EOS questionnaire

Results of the factor analysis indicated a one-factor solution for all observation scales (explained variance ranged from 55.5 to 80.2%). No factor analysis could be calculated for the observation scale on ‘degree of freedom’ at work. The Kaiser-Meyer-Olkin criterion of a bare minimum of .5 was not met, and no one-factor solution could be identified for this scale. Figure 2 shows all scales included in the STRAIN-EOS, each with its factor loadings and percentage of total variance explained.

Further results of the rotated component matrix were computed to identify which items correspond to which theoretical construct (scale), and to check for possible cross-loading of items (> 0.5) between the scales (see Table 2). Results for all scale items on demands at work (quantitative, sensorial demands, physical risks) indicate that the strongest factor loadings of each item are on the matching scale (3-factor solution with rotated factor loadings between .71–.90, 44.9–71.3% of explained variance). One item of the scale on ‘sensorial demands’ (work of the observed person demands controlling his/her movements) showed a cross-loading on the scale for ‘physical risks’ (.53); nevertheless, the strongest factor loading (.71) was on ‘sensorial demands’.

**Table 2:** Rotated component matrix (varimax) for each thematic field

	Components			
	quantitative demands	physical risks	sensorial demands	
**demands at work**^a^				
observed person (o.p.) has to work very fast	**.796**	.193	.271	
workload is unevenly distributed	**.883**	.273	−.058	
o.p. does not have time to complete all work tasks	**.828**	.164	.047	
o.p. gets behind with his/her work	**.900**	.193	.043	
o.p. does have enough time for his/her work tasks^c^	**.832**	.183	.258	
work demands very clear and precise eyesight	.036	.188	**.866**	
work demands controlling his/her movements	.089	.523	**.705**	
work demands a high level of precision	.204	−.158	**.832**	
job involves tiring or painful positions	.382	**.778**	.164	
job involves lifting or moving people	.230	**.883**	.169	
job involves carrying or moving heady loads	.192	**.853**	−.045	
**work organization and content**^a^				
	possibilities for development	influence at work		
work requires taking the initiative	**.704**	.366		
o.p. can use his/her skills or expertise	**.855**	.046		
work is varied	**.688**	.361		
work demands a high level of skill or expertise	**.868**	.038		
o.p. has a large degree of influence concerning his/her work	.242	**.720**		
o.p. has influence on who to work with	.265	**.534**		
o.p. has influence on how to do his/her work	−.006	**.784**		
o.p. has influence on what to do at work	.125	**.825**		
**social relations and leadership**^b^				
	social community	social support	social relations	predictability
o.p. is well informed in advance, e.g. about important decisions, changes or plans for the future	.080	.147	.194	**.903**
o.p. receives all information needed in order to do his/her work well	.200	.373	.202	**.742**
o.p. gets help and support from colleagues if needed	.063	**.434**	.702	.261
colleagues listen to his/her work-related problems	.197	**.581**	.571	.205
o.p. gets help and support from the immediate superior if needed	.111	**.847**	.246	.241
the immediate superior listen to his/her work-related problems	.244	**.870**	.147	.184
good atmosphere between o.p. and colleagues	**.837**	.113	.036	.141
good co-operation between o.p. and colleagues	**.863**	.238	−.016	.107
o.p. seems to be part of a community at his/her work	**.873**	.070	.098	.035
o.p. has the possibility to talk to his/her colleagues during work	.287	.327	**.692**	.232
o.p. works isolated from his/her colleagues^c^	−.130	.018	**.906**	.074

rotated component matrix on ‘demands at work’, ‘work organisation and content’ and ‘social relations and leadership’ separately, Rotation Method: Varimax with Kaiser Normalization, ^a^Rotation converged in 3 iterations, ^b^Rotation converged in 5 iterations, ^c^reversed scored item

Results for all items on ‘work organisation and content’ also revealed the strongest factor loadings of each item on the corresponding scale on ‘possibilities for development’ and ‘influence at work’ (2-factor solution, rotated factor loadings between .54–.89, 43.1–59.6% of explained variance). For these two scales, no significant cross-loading items were identified.

The further results of the rotated component matrix including all items of the scales on ‘social relations and leadership’ (predictability, social support, social community, social relations) revealed that for almost all items the highest factor loadings are on the matching scale (.43–.90, 45.5–79.6% explained variance). One item of the scale on ‘social support’ (observed person gets help and support from colleagues if needed) showed a relevant cross-loading on the scale for ‘social relations’ (.70).

### Reliability and internal consistency

The Cronbach’s alpha for the scales improved when the following items were removed. These items also had the lowest factor settings in the factor analyses.

- demands at work

- quantitative demands: “the observed person can take it easy and still do his/her work”;
- sensorial demands: “work of the observed person demands a great deal of concentration”, “work of the observed person demands constant attention”;
- physical risks: “job of the observed person involves repetitive arm or hand movements”;

b) work organisation and content

- possibilities for development: “the observed person has to do the same thing over and over again”, “the observed person has the possibility to learn new things through his/her work”, “the observed person has the opportunity to develop his/her skills through work”;
- influence at work: “other people make decisions concerning his/her work”, “the observed person has influence on how quickly to do his/her work”, “the observed person has influence on when to do his/her work”, “the observed person has influence on the amount of work”, “the observed person has influence on his/her work environment”, and “the observed person has influence on the quality of his/her work”.

As Table 3 reveals, Cronbach’s alpha is satisfactory, i.e., between .67 and .92, for the rest of the scale items included in the final STRAIN-EOS questionnaire.

**Table 3:** Properties of the final STRAIN-EOS

	N	Miss	min-max	Mdn	M (SD)	KMO	items (α)	ICC
**demands at work**								
quantitative demands	110	0	0–80	25	30 (22)	.89	5 (.92)	.74–.86
sensorial demands	109	1	17–100	67	67 (19)	.66	3 (.76)	.54–.70
physical risks	109	1	0–94	17	22 (19)	.73	3 (.88)	.72–.81
**work organization and content**								
possibilities for development	110	0	25–100	69	69 (16)	.82	4 (.83)	.52–.78
influence at work	109	1	13–88	50	51 (17)	.69	4 (.73)	.43–.60
**social relations and leadership**								
predictability	99	11	13–100	63	68 (18)	.50	2 (.67)	.50
social support	69	41	0–100	75	67 (22)	.75	4 (.87)	.68–.76
social community	108	2	42–100	83	84 (13)	.50	3 (.82)	.66–.69
social relations	110	0	0–100	50	51 (28)	.72	2 (.69)	.53

*N* Total number in sample, *Miss* Number of missing cases, *Min-max* Minimum score - maximum score, *Mdn* Median, *M* Mean, *SD* Standard deviation, *KMO* Kaiser-Meyer-Olkin criterion, number of items (Cronbach’s α), *ICC* Corrected item total correlation

### Convergent validity

Table 4 shows the additional question integrated into the external observers’ questionnaire, “How do you perceive the overall workload in the observed shift?”. Since this question addresses especially the observer’s perceived quantitative demands at work, the results revealed, as expected, the highest significant positive correlation with the scale on ‘quantitative demands’ (*r*_*τ*_ = .60, *p* < .001). Also, significant positive correlations were demonstrated for the scale on ‘physical risks’ (*r*_*τ*_ = .24, *p* < .01). Furthermore, a high workload as perceived by external observers, was negatively correlated (adverse scorings) with observation scales on work organisation and content (*p* > .05), as well as on social relations and leadership (*p* < .05). Results revealed negative correlations with observers’ overall perceived workload in the observed shift and their ratings on health professionals’ ‘predictability’ at work (*r*_*τ*_ = −.19, *p* < .05), ‘social support’ (*r*_*τ*_ = −.32, *p* < .01), ‘social community’ (*r*_*τ*_ = −.17, *p* < .05) and ‘social relations’ at work (*r*_*τ*_ = −.28, *p* < .001).

**Table 4:** Correlations of perceived stress-level of observers compared to their rating on the STRAIN-EOS

observation scale	Kendall’s tau-b	
	correlations coefficient	*p*-value
**demands at work**		
quantitative demands	.598	.000***
sensorial demands	.087	.252
physical risks	.243	.001**
**work organization and content**		
possibilities for development	−.016	.835
influence at work	−.103	.167
**social relations and leadership**		
predictability	−.191	.020*
social support	−.319	.001**
social community	−.171	.031*
social relations	−.275	.000***

*correlation is significant at: *p < .05; **p < .01; *** *p* < .001 (2-tailed)

**Fig. 3:**
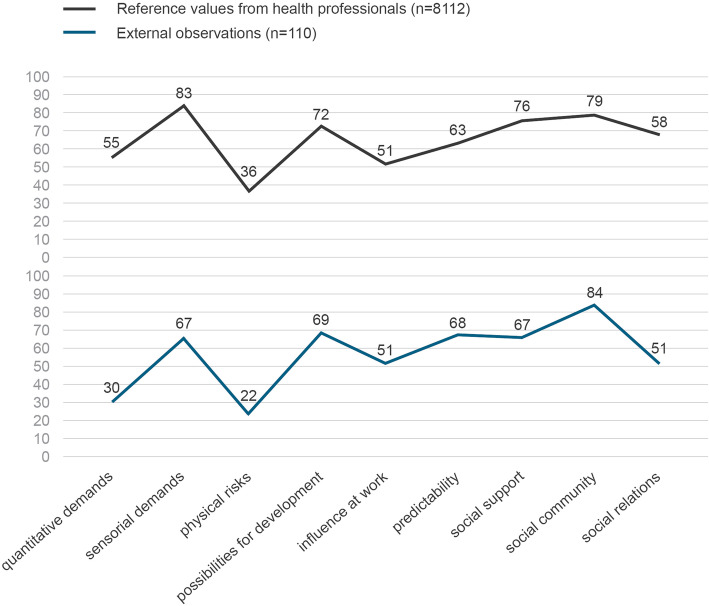
Graphic comparison of mean values from the STRAIN-EOS (n = 110 external observations) and mean values from the STRAIN questionnaire (*n* = 8′112 self-reports of health professionals) for various work stressors

In Fig. 3, mean values from the STRAIN-EOS using external observations (*n* = 110) were combined with mean values of 8112 health professionals’ self-reports on work stressors using the STRAIN questionnaire (first STRAIN measurement in 2017/2018 [24]). The self-report STRAIN questionnaire is comparable to the STRAIN-EOS, since they are based on the same underlying scales from COPSOQ [28] and EWCS [30]. Figure 3 illustrates the mean values (between 0 and 100) of self-reported (STRAIN questionnaire) and externally assessed (STRAIN-EOS assessment tool) stressors using line charts. The figure demonstrates that the mean values of stressors assessed by the STRAIN-EOS are not identical, but overall show (except for the scale on social support) similar (low or high) relative tendencies, paralleling those in the STRAIN questionnaire, which surveyed a representative sample of Swiss health professionals working in different health care settings.

### Usability

Descriptive results regarding missing values per shift of the STRAIN-EOS questionnaire, showed a low rate of missing values (< 2.7%). However, during the evening and night shifts, a higher rate of missing values was found for the scales on predictability (> 40%) and social support (> 50%), since some items were no longer observable (e.g. no colleagues or supervisor present during the night shift). According to the qualitative content analysis of the comments from the external observers, all questions were understandable. A total of 43 written comments from external observers were analysed. Most reasons declared by external observers for non-observable items were that the observed persons had “no contact with supervisors” during the observed shift (29 comments) or that a certain situation did not occur (e.g., the question on predictability ‘At your place of work, are you informed well in advance concerning for example important decisions, changes, or plans for the future?’) and, therefore, was not observable during the shift (12 comments).

## Discussion

The results of this study revealed that 9 of the 10 tested observation scales were internally consistent, had good content and construct validity, and to some extent, convergent validity as well as sufficient usability. However, the results also demonstrated that the STRAIN-EOS questionnaire needs further testing, for example regarding the convergent validity with other observation-based assessment tools or interobserver reliability.

In regard to the observation scales on the ‘degree of freedom at work’, no factor analysis could be calculated. Since one item from the self-assessment scale, “Can the observed person take holidays when he/she wishes?”, was not observable during one shift only, the observation scale on degree of freedom consisted of 3 instead of 4 items. It is conceivable that the observation scale will show construct validity if this item is replaced.

Further results on the cross-loading of items gives confirmation of the construct validity and reveals that all items, except one, in the STRAIN-EOS had their strongest loading on the matching scale. The item of the scale on ‘social support’ showed a cross-loading on the scale ‘social relations. This can be explained by the fact that the item ‘the observed person gets help and support from colleagues if needed’ cannot be easily separated from the scale of ‘social relations’ at work in terms of content. However, if the STRAIN-EOS is used again, this should be checked again with a new sample.

Furthermore, comparisons between overall perceived workload as rated by observers and their ratings in the STRAIN-EOS reveal several significant correlations in the expected positive or negative direction, but also some weak correlations. The convergence between health professionals’ self-reported and externally assessed stressors regarding the same shift was investigated in a separate study [34] and showed convergent scores for 3 of the 9 tested scales. This low convergence when comparing different methods for assessing work stressors is also described in previous literature [10]. Therefore, a further analysis of correlations between the STRAIN-EOS and another validated external observation questionnaire assessing stressors at work could further strengthen the convergent and construct validities of the newly developed instrument.

### Limitations

Despite the extensive training of external observers, a possible observer bias cannot be excluded, since all observers differ in age, memories, behaviour and professional experience. Moreover, the STRAIN-EOS was tested in several healthcare settings that included different health professionals. Since a convenience sampling strategy was used, the distribution of observations in the settings, shifts and health professions was not controlled. Therefore, the extent to which this observation-based assessment can be generalized to other settings or specific professions is limited and further research is clearly needed to make a specific statement about the use of this instrument for one specific professional group (e.g. physiotherapists only) or setting (e.g. nursing homes) only. Moreover, the STRAIN-EOS was developed in German and needs to be translated and tested before use in other languages.

### Methodological challenges and limitations

A difficulty when using external observations in the healthcare setting was to measure interobserver reliability. On the one hand, patients did not accept having more than one external observer accompany the health professional; on the other hand, this was not possible for organisational reasons (e.g. hygiene guidelines, isolated patients, confined space conditions, disruption of workflows). Consequently, we decided to test interobserver reliability using video sequences. As the results show however, this was not entirely feasible either. Regarding the results of the partly unsatisfactory ICC results assessed by video sequences, it is difficult to draw conclusions. On the one hand, it is quite possible that the video sequences were not suitable, for example, to observe ‘predictability’. On the other hand, it is also possible that the observers` ratings in the STRAIN-EOS strongly diverged from one another. Therefore, we recommend taking into account possible observer effects when analysing the STRIAN-EOS scales (e.g. by using multiple regression analysis in which different observers are included as dummy variables). Moreover, further research is also needed regarding interobserver reliability using the STRAIN-EOS.

Another difficulty for external observers could be not knowing how to behave in certain cases, e.g. patient aggression against the observed health professional, observed violations of safety measures, unexpected deaths of patients or emergency situations such as reanimation. For this reason, we decided on having external observers with a professional background in the healthcare sector, because they are already familiar with such situations and can deal with them. In addition, we defined a code of conduct for all external observers as to how they should behave in such emergencies (e.g. assistance with reanimation).

A further difficulty was the 24-h operation of health institutions, observations of evening and night shifts and observed health professionals doing overtime. In order for the external observers to observe evening and night shifts, a special employment contract was drawn up that allowed them to work in those shifts and settled the payment for it. In addition, it was agreed with the external observers that the observation would be continued even if the observed health professional worked overtime, provided it did not exceed 1–2 h. This could be observed, except for one observation in which the observed health professional worked longer than 2 h overtime.

Data protection and privacy of patients was also a special topic when doing external observation in the healthcare sector. The external observers were encouraged to maintain silence on patient-specific data. In addition, patients and professionals had the right at any time to send the observed person out of the room in very personal situations (e.g. when inserting a bladder catheter). Also, no patient-specific data were recorded in the STRAIN-EOS either.

## Conclusions

As the results of the study reveal, the use of external observers in a healthcare system does not occur without effort. However, as the results of the study show, it is important to have a suitable observation tool specifically tailored to the requirements and framework conditions in healthcare. The STRAIN-EOS developed in this study is a first step but needs further testing. Also, research is needed to investigate the interrelationship between health professionals’ self-reports and objective results derived from observation-based assessment tools. With the development of the STRAIN-EOS based on validated and reliable self-assessment scales, such a comparison is conceivable in the future. This will assist in gaining deeper knowledge of the issues concerning work-related stressors in health professions.

## Data Availability

Raw data was generated at the Bern University of Applied Sciences, Department of Nursing Research. Derived data supporting the findings of this study is available from the corresponding author (karin.peter@bfh.ch) upon request. The STRAIN-EOS questionnaire is also available upon request from the corresponding author.

## Abbreviations

COPSOQ: Copenhagen Psychosocial Questionnaire
EWCS: European Working Conditions Survey
LEP: Leistungserfassung in der Pflege
o.p.: Observed person
STRAIN: Work-related stress among health professionals in Switzerland
STRAIN-EOS: STRAIN - External Observation of work Stressors

## References

[1] Eurofound (2019). Working conditions and workers’ health. Luxembourg: Publications Office of the European Union.

[2] Lim J, Bogossian F, Ahern K (2010). Stress and coping in Australian nurses: a systematic review. Int Nurs Rev.

[3] Fuss I, Nubling M, Hasselhorn HM, Schwappach D, Rieger MA (2008). Working conditions and work-family conflict in German hospital physicians: psychosocial and organisational predictors and consequences. BMC Public Health.

[4] Hämmig O (2018). Explaining burnout and the intention to leave the profession among health professionals - a cross-sectional study in a hospital setting in Switzerland. BMC Health Serv Res.

[5] Leka S, Jain A (2010). Health impact of psychosocial hazards at work: an overview.

[6] Long MH, Johnston V, Bogossian F (2012). Work-related upper quadrant musculoskeletal disorders in midwives, nurses and physicians: a systematic review of risk factors and functional consequences. Appl Ergon.

[7] Aiken LH, Clarke SP, Sloane DM, Sochalski J, Silber JH (2002). Hospital nurse staffing and patient mortality, nurse burnout, and job dissatisfaction. J Am Med Assoc.

[8] Lancet T (2019). Physician burnout: a global crisis. Lancet.

[9] Lee R, Seo B, Hladkyj S, Lovell BL, Schwartzmann L (2013). Correlates of physician burnout across regions and specialties: a meta-analysis. Hum Resour Health.

[10] Semmer NK, Grebner S, Elfering A, Perrewe PL, Ganster DC (2004). Beyond self-report: Using observational, physiological, and situation-based measures in research on occupational stress. Research in Occupational Stress and Well Being.

[11] Kompier M (2005). Assessing the psychosocial work environment—“subjective” versus “objective” measurement. Scand J Work Environ Health.

[12] Connell P, Lee VB, Spector PE, Thomas JC, Hersen M (2004). Job stress assessment methods. Comprehensive handbook of psychological assessment: Industrial and organizational assessment.

[13] Corradini I, Marano A, Nardelli E (2016). Work-related stress risk assessment: a methodological analysis based on psychometric principles of an objective tool. SAGE Open.

[14] Frese M, Zapf D, Cooper CL, Payne R (1988). Methodological issues in the study of work stress: Objective vs. subjective measurement of work stress and the question of longitudinal studies. Causes, coping and consequences of stress at work.

[15] Foster P, Sapsford R, Jupp V (2006). Observational Research. Data Collection and Analysis. Volume 2.

[16] Monahan T, Fisher JA (2010). Benefits of “observer effects”: lessons from the field. Qual Res.

[17] Hull L, Arora S, Kassab E, Kneebone R, Sevdalis N (2011). Assessment of stress and teamwork in the operating room: an exploratory study. Am J Surg.

[18] Syed I, Daly T, Armstrong P, Lowndes R, Chadoin M, Naidoo V (2016). How do work hierarchies and strict divisions of labour impact care workers’ experiences of health and safety? Case studies of long term care in Toronto. J Nurs Home Res.

[19] Stab N, Hacker W, Weigl M (2016). Work organization in hospital wards and nurses’ emotional exhaustion: a multi-method study of observation-based assessment and nurses’ self-reports. Int J Nurs Stud.

[20] Wheelock A, Suliman A, Wharton R, Babu ED, Hull L, Vincent C, Sevdalis N, Arora S (2015). The impact of operating room distractions on stress, workload, and teamwork. Ann Surg.

[21] Büssing A, Glaser J (2002). Das Tätigkeits-und Arbeitsanalyseverfahren für das Krankenhaus-Selbstbeobachtungsversion (TAA-KH-S): Hogrefe.

[22] Weigl M, Muller A, Zupanc A, Angerer P (2009). Participant observation of time allocation, direct patient contact and simultaneous activities in hospital physicians. BMC Health Serv Res.

[23] Golz C, Peter KA, Hahn S (2018). Cognitive pretesting and pretest of the STRAIN questionnaire to elaborate work-related stress of health care staff in Switzerland. Int J Health Professions.

[24] Peter KA, Schols JMGA, Halfens RJG, Hahn S (2020). Investigating work-related stress among health professionals at different hierarchical levels: a cross-sectional study. Nurs Open.

[25] Spector PE, Dwyer DJ, Jex SM (1988). Relation of job stressors to affective, health, and performance outcomes: a comparison of multiple data sources. J Appl Psychol.

[26] Kristensen TS (2000). A new tool for assessing psychosocial factors at work: the Copenhagen psychosocial questionnaire.

[27] Nübling M, Stössel U, Hasselhorn HM, Michaelis M, Hofmann F (2006). Measuring psychological stress and strain at work: evaluation of the COPSOQ questionnaire in Germany. GMS Psycho-Social-Medicine.

[28] Nübling M, Vomstein M, Nolle I, Lindner A, Haug A, Lincke HJ (2017). Deutsche Standard-Version des COPSOQ 2017.

[29] Nübling M, Stößel U, Hasselhorn HM, Michaelis M, Hofmann F (2005). Methoden zur Erfassung psychischer Belastungen: Erprobung eines Messinstruments (COPSOQ).

[30] Eurofound (2015). Sixth European Working Conditions Survey - Questionnaire.

[31] Parent-Thirion A, Fernández Macías E, Hurley J, Vermeylen G (2007). Fourth European working conditions survey.

[32] LEP-AG (2017). Methode zur Dokumentation und Auswertung von Leistungen im Gesundheitswesen.

[33] Shrout PE, Fleiss JL (1979). Intraclass correlations: uses in assessing rater reliability. Psychol Bull.

[34] Peter KA, Hahn S, Stadelmann E, Halfens R, Schols J (2020). Assessing work stressors in the health care sector by combining external observation and health professionals’ self-report in a cross-sectional study design. Occup Med Health Affairs.

